# Shengji ointment accelerates diabetic foot ulcer wound healing by regulating inflammatory microenvironment

**DOI:** 10.3389/fphar.2026.1855791

**Published:** 2026-09-03

**Authors:** Rui Dai, Jing Guo, Jinpeng Jing, YunSha Zhang, HongBin Liu, Zhaohui Zhang, Chaojun Zhu

**Affiliations:** 1 Graduate School, Tianjin University of Traditional Chinese Medicine, Tianjin, China; 2 Institute of TCM Ulcers, Tianjin University of Traditional Chinese Medicine, Tianjin, China; 3 Department of Traditional Chinese Surgery, Second Affiliated Hospital of Tianjin University of Traditional Chinese Medicine, Tianjin, China; 4 Department of Peripheral Vascular Diseases, Shandong University of Traditional Chinese Medicine Affiliated Hospital, Jinan, China; 5 School of Integrative Medicine, Tianjin University of Traditional Chinese Medicine, Tianjin, China; 6 Heping District Health Commission, Tianjin, China

**Keywords:** diabetic foot ulcers, inflammatory microenvironment, Shengji ointment, Traditional Chinese Medicine, wound healing

## Abstract

**Background:**

Diabetic foot ulcers (DFUs) represent a significant clinical challenge due to their high morbidity, infection risk, and poor healing rates. Emerging evidence highlights the therapeutic potential of Traditional Chinese Medicine in wound management. This study investigates the efficacy of Shengji Ointment (SJ)—a multi-herb formulation—in human subjects with DFUs and a diabetic rat model, specifically elucidating its modulation of the wound inflammatory microenvironment.

**Methods:**

The lyophilized powder of the SJ extract was analyzed using UPLC-MS/MS to profile its chemical constituents. A single-center, prospective, randomized, controlled clinical study was conducted in patients with Wagner Grade 3 DFUs. A diabetic wound rat model was established, and proinflammatory cytokine and growth factor levels in granulation tissue were quantified at multiple time points.

**Results:**

UPLC-MS/MS identified 3,552 distinct UPLC-MS/MS identified 3,552 distinct compounds in the SJ extract, predominantly flavonoids and alkaloids in the SJ extract, predominantly flavonoids and alkaloids; 10 high-abundance compounds were selected (5 at MSI Level 1). The clinical study demonstrated that SJ significantly accelerated DFU wound healing. The animal study revealed that SJ promotes wound healing by modulating the wound microenvironment.

**Conclusions:**

This study demonstrated that Shengji Ointment (SJ) could inhibit excessive inflammation, promote angiogenesis and vascular maturation, and thereby markedly accelerate diabetic wound healing in both clinical settings and rat models. Such therapeutic effects may be associated with the DLL4/Notch1-VEGF/VEGFR2 signaling pathway.

## 1 Introduction

The global prevalence of diabetes mellitus (DM) is projected to reach 10.2% of the world’s population by 2030 and will affect 700 million people by 2045, driven by economic development and lifestyle changes (Saeedi et al., 2019). Diabetic foot ulcers (DFUs) are a severe complication, characterized by a high incidence, amputation, recurrence, and mortality. Epidemiological data show that the 5-year mortality rate for patients with DFUs is approximately 30%. The 5-year mortality rate among patients who undergo major amputation exceeds 70%. Evidence suggests that between 30% and 40% of DFUs heal within 12 weeks (Armstrong et al., 2023). However, the DFU recurrence rate can reach up to 65% within 5 years (McDermott et al., 2023). The management of DFU typically requires extended, ongoing treatment and rehabilitation, resulting in a substantial financial burden for patients and families. Furthermore, DFU patients experience uncertainty about disease progression and recurrence risk, frequently leading to anxiety, depression, and other psychological comorbidities. Consequently, developing effective medical strategies to promote DFU wound healing represents an urgent clinical priority requiring immediate surgical attention.

Chronic overproduction of proinflammatory cytokines disrupts wound healing processes, including angiogenesis impairment, micro vessel and macro vessel dysfunction, altered macrophage polarization, and wound healing mediator deficiency (Ahmed et al., 2026; Dawi et al., 2025). Aberrant macrophage polarization has been shown to result in excessive proinflammatory factor production, tissue-repair growth factor deficiency, angiogenic factor insufficiency, and an imbalance between vascular sprouting and maturation, which contributes to the therapeutic recalcitrance of diabetic wounds (Lee et al., 2018; Nirenjen et al., 2023). Vascular endothelial growth factor (VEGF) signaling is the most well-characterized angiogenic pathway, serving as the principal regulator of both physiological and pathological blood vessel formation. Vascular endothelial growth factor-A (VEGF-A) is the predominant and most potent angiogenic factor, commonly referred to simply as “VEGF.” There are three subtypes of VEGF receptors: VEGFR1, VEGFR2, which are primarily expressed on vascular endothelial cells, and VEGFR3, which is localized to the lymphatic system in healthy adults (Johnson and Wilgus, 2014; Zhang et al., 2015). VEGF and Notch signaling collaborate to regulate angiogenesis in wound healing.

Notch signaling is an evolutionary conserved pathway in multicellular organisms that regulates cell fate during development and homeostasis maintenance in adult tissues. This pathway serves as a critical regulator of wound healing processes, including inflammation, vascularization, epithelialization, and scar formation. Furthermore, it mediates the selective recruitment of vascular endothelial cells, orchestrates the morphogenesis and functional maturation of blood vessels, and facilitates vascular regeneration following tissue injury (Koon et al., 2018; He et al., 2020). Notch signaling pathway consists of four receptor subtypes (Notch1-4) and five ligands (Jagged1-2, Delta-like (DLL)1/3/4). Beyond its well-known role in angiogenesis, this pathway serves as a downstream target of inflammatory mediators, a key regulatory system controlling the balance between M1 and M2 macrophage polarization (Barman and Koh, 2020). Current evidence indicates that inflammation triggers the Notch signaling pathway activation, macrophage polarization, and macrophage-mediated release of inflammatory factors. Notch signaling has emerged as a promising therapeutic target for suppressing inflammatory responses (He et al., 2020).

Some Traditional Chinese Medicine preparations (TCM) and its key phytochemicals exhibit therapeutic potential for diverse pathological conditions. Extensive clinical and experimental studies have substantiated their multi-target mechanisms in modulating inflammatory response (Mao et al., 2024; Wu et al., 2025), improving microcirculation (Wang et al., 2022; Li Y. et al., 2025), regulating cellular differentiation (Aitcheson et al., 2021; Li M.C. et al., 2024), promoting tissue repair (Tan et al., 2024; Feng et al., 2025; Zhu et al., 2019),and anticancer mechanisms (Faida et al., 2024; Li Z. et al., 2024; Hua and Bergers, 2019; Kong et al., 2024). Shengji Ointment (SJ) is a hospital-prepared traditional Chinese medicinal formulation composed of 10 medicinal plants (in Section 2.1). Its clinical efficacy in wound healing has been documented in previous studies (Sun et al., 2024). However, the chemical basis underlying its pharmacological effects remains incompletely characterized. Angelica dahurica is primarily employed, which exerts anti-inflammatory effects and promotes pus clearance. Phytochemical analysis reveals the chemical compounds profile is dominated by coumarins, followed by a variety of other chemical compounds, including glycosides, alkaloids, polysaccharides, and amino acids (Zhao et al., 2022; Zhang et al., 2017). Ligusticum chuanxiong contains chemical compounds such as phenyl pyridine derivatives (*>*45%), organic phenolic acids, alkaloids, and a small number of polysaccharides. Modern pharmacological research has confirmed its systemic protective effects, including anti-ischemia, anti-inflammation, pro-angiogenesis, and anti-tumor (Liu et al., 2022; Thanh et al., 2022; Liu et al., 2018). Rehmannia glutinosa contains multiple key chemical compounds such as anthraquinones, rolandones, triterpenoids, cycloartenols, flavonoids, and sugars (Wang et al., 2026), which can inhibit various bacteria such as *Escherichia coli* and *Staphylococcus aureus*. Therefore, it has anti-inflammatory properties (Lu et al., 2022; Wang et al., 2025). Angelica sinensis contains polysaccharides, ferulic acid, and ligustilide, which are implicated in hematopoietic modulation, regulation of the menstrual cycle, resolution of blood stasis, and analgesia. It also contains vitamins and lactones that have been shown to potently inhibit inflammatory factor release, enhance local blood circulation, and accelerate wound healing (Wei et al., 2016; Nai et al., 2021). *Lonicera japonica*, the dried flower bud of *L. japonica*, contains active components of chlorogenic acid, flavonoids, triterpenoid saponins, and essential oils (linalool, geraniol), which exhibit antibacterial, anti-inflammatory, antioxidant, anti-thrombotic, and immune-modulating actions. Traditionally, it was used to clear heat, detoxify, and dispel wind-heat (Zhou et al., 2022; Liu et al., 2019; Lv et al., 2021).

This study aims to confirm the clinical safety and efficacy of SJ in treating diabetic wounds. Furthermore, the mechanisms in regulating wound microenvironment, such as inflammatory responses and angiogenesis, were investigated to elucidate the wound-healing properties.

## 2 Materials and methods

### 2.1 UPLC-MS/MS-based phytochemical profiling of the SJ lyophilized powder derived from the ointment

Shengji Ointment (SJ) is a traditional Chinese medicinal ointment formulated with 10 medicinal plants at fixed dosages. *Angelica dahurica* (Fisch. ex Hoffm.) Benth. et Hook. f. ex Franch. et Sav. 100 g; *Ligusticum* chuanxiong Hort. 100 g; *Angelica sinensis* (Oliv.) Diels 90 g; *Rehmannia glutinosa* (Gaertn.) Libosch. ex Fisch. et Mey. 90 g; *L. japonica* Thunb. 60 g; *Sanguisorba officinalis* L. 60 g; *Arnebia euchroma* (Royle) Johnst. 30 g; *Ampelopsis japonica* (Thunb.) Makino. 30 g; *Coptis chinensis* Franch. 30 g; *Phellodendron chinense* Schneid. 15 g. Beeswax was adopted as the pharmaceutical excipient (ratio of crude herbs to dried extract: 4:1; ratio of dried extract to final ointment: 23:100). The final ointment presented as a brown, homogeneous preparation with a pH value ranging from 5.5 to 7.0, uniform particle size, and compliant microbial limits. All the above-mentioned crude drugs were uniformly purchased and prepared by the Pharmacy Department of the Second Affiliated Hospital of Tianjin University of Traditional Chinese Medicine (hospital preparation registration number: Z20070652; batch number of the preparation used in this study: 2001002). Given the complexity of the multi-herb formulation and the semi-solid ointment matrix, UPLC-MS/MS-based phytochemical profiling was employed to comprehensively characterize the chemical composition of SJ and identify its major bioactive constituent classes.

For UPLC-MS/MS analysis, the beeswax excipient was first removed from the SJ ointment to obtain the active fraction. Briefly, the ointment was heated at 80 °C to melt the beeswax, which was then separated by centrifugation at 10,000 rpm for 10 min. The resulting active fraction was then extracted with anhydrous methanol under ultrasonication for 30 min at room temperature. The extract was subsequently concentrated under reduced pressure and lyophilized using a freeze-dryer (Scientz-100F) for 63 h to obtain the SJ lyophilized powder. 50 mg of SJ powder was mixed with 1,200 μL of pre-cooled (20 °C) 70% methanol/water(v/v). The mixture was vortexed every 30 min over a total of 3 h (6 cycles, 30 s each). The supernatant was collected after centrifugation (12,000 rpm, 3 min), filtered through a 0.22 μm microporous membrane, and subjected to UPLC-MS/MS analysis. For the purpose of phytochemicals qualification and quantitative validation, a panel of reference standards was independently prepared by dissolving each standard in 70% methanol/water to obtain stock solutions at a concentration of 250 μg/mL. These reference standards corresponded to representative compounds from the major chemical classes identified in the SJ extract, including selected phenolic acids, alkaloids, and lactones. These reference standards were analyzed under the identical UPLC-MS/MS conditions to confirm their retention times and MS/MS spectra for subsequent comparison with the detected features in the SJ samples.

Chromatographic conditions include: (1) Column: Agilent SB-C18 (1.8 µm, 2.1 mm × 100 mm); (2) Mobile phase: Phase A: ultrapure water with 0.1% formic acid, Phase B: acetonitrile with 0.1% formic acid; (3) Elution gradient. 0.00 min: 5% B, 0.00–9.00 min: linear increase to 95%, 9.00–10.00: hold 95% B, 10.00–11.10 min: decrease to 5% B, and 11.00–14.00 min: re-equilibrate at 5% B; (4) Flow rate 0.35 mL/min; (5) Column temperature 40 °C; (6) Injection volume 2 μL.

Mass spectrometry conditions include: (1) Ionization model: electrospray ionization (ESI) (2) Ion source parameters: temperature 500 °C, ion spray voltage (IS) 5500 V (positive)/−4500 V (negative), gas flow (ion source gas I 50, gas II 60, and curtain gas 25 psi), and collision gas (nitrogen) medium; (3) Detection mode: multiple reaction monitoring (MRM); (4) Optimized parameters: declustering potential (DP) and collision energy (CE); (5) Dynamic MRM: specific ion pairs based on the metabolites eluted windows.

Compounds identification was performed by matching the acquired MS/MS spectral data against the in-house MetWare database (MWDB). Before analysis, isotope signals, adducts (K+, Na+, NH4+), and fragment ions corresponding to higher molecular weight compounds were systematically removed to ensure data accuracy.

### 2.2 Clinical study

This study was approved by the Ethics Committee of the Second Affiliated Hospital of Tianjin University of Traditional Chinese Medicine (Ethics Approval Number: 2020-006-01),The study protocol was registered in the Chinese Clinical Trial Registry (ChiCTR) under the code ChiCTR2000039327 on 23 October 2020. Based on previous findings, the wound healing rate of the control group was 63% after 4 weeks of treatment. A 20% increase in healing rate was anticipated for the experimental group. The significance level α was set to 0.05, and the statistical power was set to 90%. Sample sizes were equal between the two groups. The sample size was calculated using the formula for comparison of two sample rates:

$$n=\frac{{\left(Z_{1-\alpha /2}+Z_{1-\beta }\right)}^{2}*\left[p1\left(1-p1\right)+p2\left(1-p2\right)\right]}{{\left(p2-p1\right)}^{2}}$$

In this formula, *n* stands for the required number of cases in each group. Z_1−α/2_ and Z_1−β_ represent the standard normal deviates. *p1* is the healing rate of the control group, and *p2* refers to the expected healing rate of the experimental group. Patients (total patients n = 104, SJ group and control group at a 1:1 ratio, 18–80 years old, no gender restrictions) with one or more Wagner Grade 3 DFUs (total ulcers n = 104, size range 1–20 cm^2^) were enrolled, meeting the established diagnostic criteria (Rai, Moellmer, and Agrawal, 2022). Following debridement, patients were randomly divided into two groups.

The SJ group received daily applications of an approximately 0.1 cm-thick SJ dressing (0.1 g/cm^2^), daily rbFGF dressings were applied to the control group. Debridement was carried out prior to each dressing change. Normal saline and sterile instruments were prepared before the procedure, and the animals were properly fixed. The dressings were gently removed. For the adhered parts, moisten them with normal saline and then peel them off. Wipe the wound surface from the inside out with normal saline to remove secretions, loose scabs and foreign objects. To avoid the interference of other drugs, use normal saline to wipe the wound surface in a circular motion from the center of the wound to the outside, with the wiping range extending more than 0.5 cm beyond the wound surface. After natural air - drying, dip up the residual liquid. For fresh wounds, the main focus is on cleaning and protection. No sharp debridement is performed on necrotic tissues. After the operation is completed, apply medicine and bandage the wound in a timely manner. The rbFGF was sprayed directly onto wounds, or evenly sprayed onto sterile gauze covering lesions without liquid overflow, followed by proper dressing. The dosage was 262.5iu per square centimeter once daily. The wounds were assessed at 2 and 4 weeks after treatment. Blinding was not feasible in this study. The conventional surgical dressing protocol for the control group and the external traditional Chinese medicine therapy for the experimental group differed greatly in dressing procedures and drug appearance. This study adopted an open-label design, and outcome evaluations were conducted by an independent third party to reduce bias.

#### 2.2.1 Assessment of wound healing

Wound areas were quantified at baseline (S0), and 2- and 4-week post-treatment (Sn) using the Kushite Ekare Insight 3D Wound Measurement and Recording System (V1.7.5; Beijing Daxiong Weiyue Pharmaceutical Technology). Wound healing rate was calculated as: [(S0 − Sn)/S0] × 100%.

Epithelial proliferation was scored at 2- and 4-week post-treatment using standard criteria (see appendix). Wounds presenting contraction were marked as “partially or fully healed,” while wounds without contraction or with expansion were marked as “unhealed.”

#### 2.2.2 Determination of serum angiogenic factors by ELISA

Peripheral blood samples were collected at baseline and 4 weeks post-treatment following written informed consent. Serum was isolated after centrifugation at 2000 rpm for 25 min and stored at −80 °C until analysis. Serum concentrations of VEGF, VEGFR2, Notch1, and DLL4 were quantified using ELISA kits according to manufacturers’ protocols.

### 2.3 Experimental animal study

#### 2.3.1 Establishment of diabetic rat wound model

Male Sprague-Dawley rats (specific pathogen-free [SPF]; n = 90; age: 6 weeks; weight: 180–200 g) were used in this study. All experimental procedures were approved by the Ethics Review Committee of the Institute of Radiation Medicine, Chinese Academy of Medical Sciences (Approval No. IRM-DWLL-2022127). Rats were acclimatized for 7 days, followed by a 12-h fast with free access to water before diabetes induction.

The diabetes was induced by intraperitoneal injection of 1% streptozotocin (STZ) solution (40 mg/kg) for two consecutive days (Furman, 2021; Rai et al., 2022). Seventy-two hours after the final STZ injection, non-fasting blood glucose levels were measured, with levels ≥16.7 mmol/L confirming diabetic status.

**FIGURE 1:**
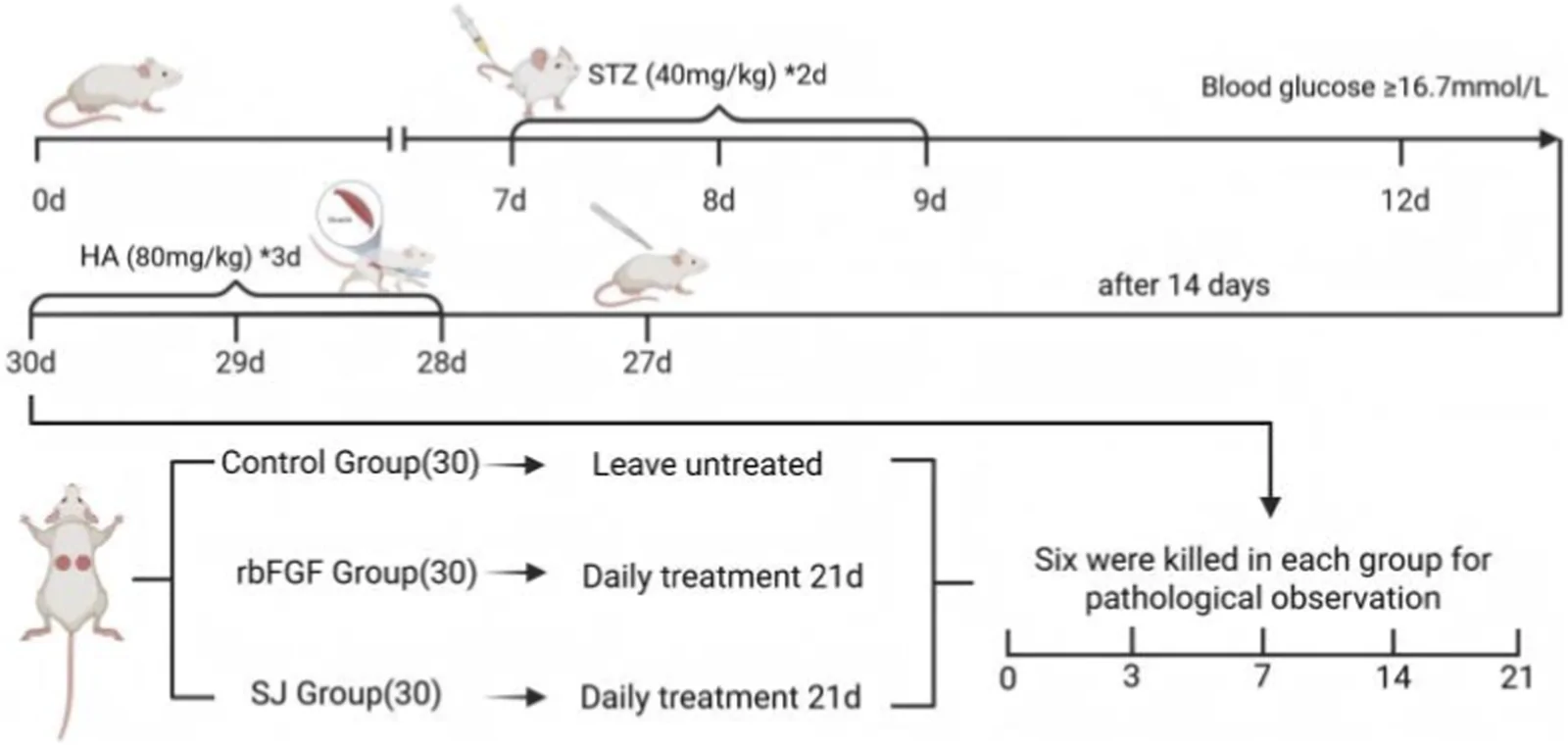
A flowchart shows the timeline of model establishment, wound treatment, and biopsy collection.

Fourteen days after successful diabetes induction, two full-thickness excision wounds (diameter: 2 cm) were created bilaterally on the dorsal surface (1 cm apart from the midline). Rats subsequently received intramuscular injection of hydrocortisone acetate (80 mg/kg) for three consecutive days (Jozic et al., 2021). Subsequently, 90 rats were randomly divided into three groups: the control group (without any intervention), the rbFGF group (using rbFGF film), and the SJ group (using SJ film), with 30 rats in each group. During the 21 days after injury, all dressings were changed once a day, and six rats from each group were sacrificed at 0, 3, 7, 14, and 21 days for pathological and histological observations (Figure 1). No animal deaths or infections occurred during the experimental period.

#### 2.3.2 Wound measurement

Wound margins were traced onto transparent paper. The tracings were then excised and weighed. Wound area was calculated as: weight of wound tracing/weight of 1 cm^2^ transparent paper.

Healing rate was calculated as: [(initial wound area–current unhealed wound area)/initial wound area] × 100%.

Healing time was recorded as the number of days (d) from the treatment initiation to complete epithelialization.

#### 2.3.3 Collection of wound biopsies

Wound biopsies were collected on days 3, 7, 14, and 21 post-treatments under anesthesia, including the wound margin and granulation tissue for histological staining and real-time PCR.

### 2.4 IHC

Paraffin sections were routinely deparaffinized in xylene and rehydrated through a graded ethanol series (100%, 95%, 80%, 70%) to distilled water. For HE staining, hematoxylin was used to stain the nuclei, and eosin was used to stain the cytoplasm. For Masson trichrome staining, sections were stained with Weigert iron hematoxylin, Masson blue, and carmine solutions. After dehydration, sections were mounted with neutral resin.

The collagen content in Masson’s trichrome-stained sections was quantified using ImageJ (Fiji) with the color deconvolution plugin. Five non-overlapping high-power fields were randomly selected per sample. The blue-stained collagen area was segmented by uniform threshold, and the collagen volume fraction (CVF) was calculated as the ratio of collagen area to total tissue area. All quantitative parameters were kept consistent across all groups.

### 2.5 ELISA

Wound biopsies were cut into small pieces, and 50 mg tissue was homogenized in 500 μL lysis buffer with PMSF (1 mM). Then the lysis was incubated on ice for 30 min and centrifuged at 12,000 g for 15 min. Collected the supernatant, and IL-6, 10, TNF-α, TGF-β, MMP-2, MMP-9, VEGF, VEGFR2, DLL4, Notch1 were detected according to the instructions of the ELISA kits by microplate reader (Manufacturer: TECAN, Model: Infinite E Plex).

### 2.6 Real-time PCR

Total RNA was extracted from wound tissues using Trizol reagent (Invitrogen, Carlsbad, CA, United States 15596026). First stand cDNA was synthesized from total RNA using a reverse transcription kit (Kangwei Century CW2582). Gene expressions of IL–6, IL–10, TNF-a, TGF-β, VEGF, VEGFR2, DLL4, and Notch1 were quantified using iTaq Universal SYBR Green Supermix (Bio-Rad, Hercules, CA, United States) by real time QUANTITATIVE PCR (PCR) on an ABI 7500 Fast Real-Time PCR System (Applied Biosystems, Foster, CA, United States). The thermal cycling conditions are 95 °C for 10 min; 40 cycles of 95 °C for 15 s, 60 °C annealing for 1 min, and 72 °C extension for 30 s. Rat actin was used as an endogenous control. All reactions were performed in technical triplicate, and results are presented as mean fold (2^−△△^CT). All primer sequences are provided in Table 1.

**TABLE 1:** Primer sequences.

Primer name	Sequences (5′-3′)
IL-6	F-GTTGCCTTCTTGGGACTGATG R-TACTGGTCTGTTGTGGGTGGT
IL-10	F-TGCTATGTTGCCTGCTCT R-AGTGGGTGCAGTTATTGTC
TNF-α	F-GCATGATCCGAGATGTGGAACTGG R-CGCCACGAGCAGGAATGAGAAG
TGF-β	F-CTCCCGTGGCTTCTAGTGC R-GCCTTAGTTTGGACAGGATCTG
VEGF	F-GTCACCACCACACCACCATCGT R-CTCCTCTCCCTTCATGTCAGGCT
VEGFR-2	F-AAGAGATTTGTTCCGGATGG R-CGGCAGATAGCTCAATTTCA
DLL4	F-GCGGATAACCAACGACG R-CAAGCCCACGGGAAACT
Notch1	F-GATTGATGTCAACGAGTG R-CTCATAACCTGGCATACA
Actin	F-CACCCGCGAGTACAACCTTC R-CCCATACCCACCATCACACC

### 2.7 Statistical analysis

Statistical analysis was performed using SPSS 22.0 and GraphPad 10.0 Prism software. The signific Continuous variables were first assessed for normality via the Shapiro–Wilk test. Normally distributed measurement data were expressed as mean ± standard error of the mean (SEM), while non-normally distributed data were described as median (P25, P75) and analyzed with corresponding non-parametric statistical tests. Categorical data were compared using the chi-square test. For intergroup comparisons of normally distributed quantitative data, one-way analysis of variance (ANOVA) was adopted, and repeated-measures ANOVA was used for intra-group comparisons of repeatedly measured indicators. Correlation analyses were conducted to evaluate monotonic associations between research variables, Whereas Spearman’s rank correlation analysis was performed for non-normally distributed data. All statistical tests were two-tailed with a significance level set at α = 0.05, *P < 0.05* indicated statistical significance.

## 3 Results

### 3.1 Chemical constituents of the SJ extract

**FIGURE 2:**
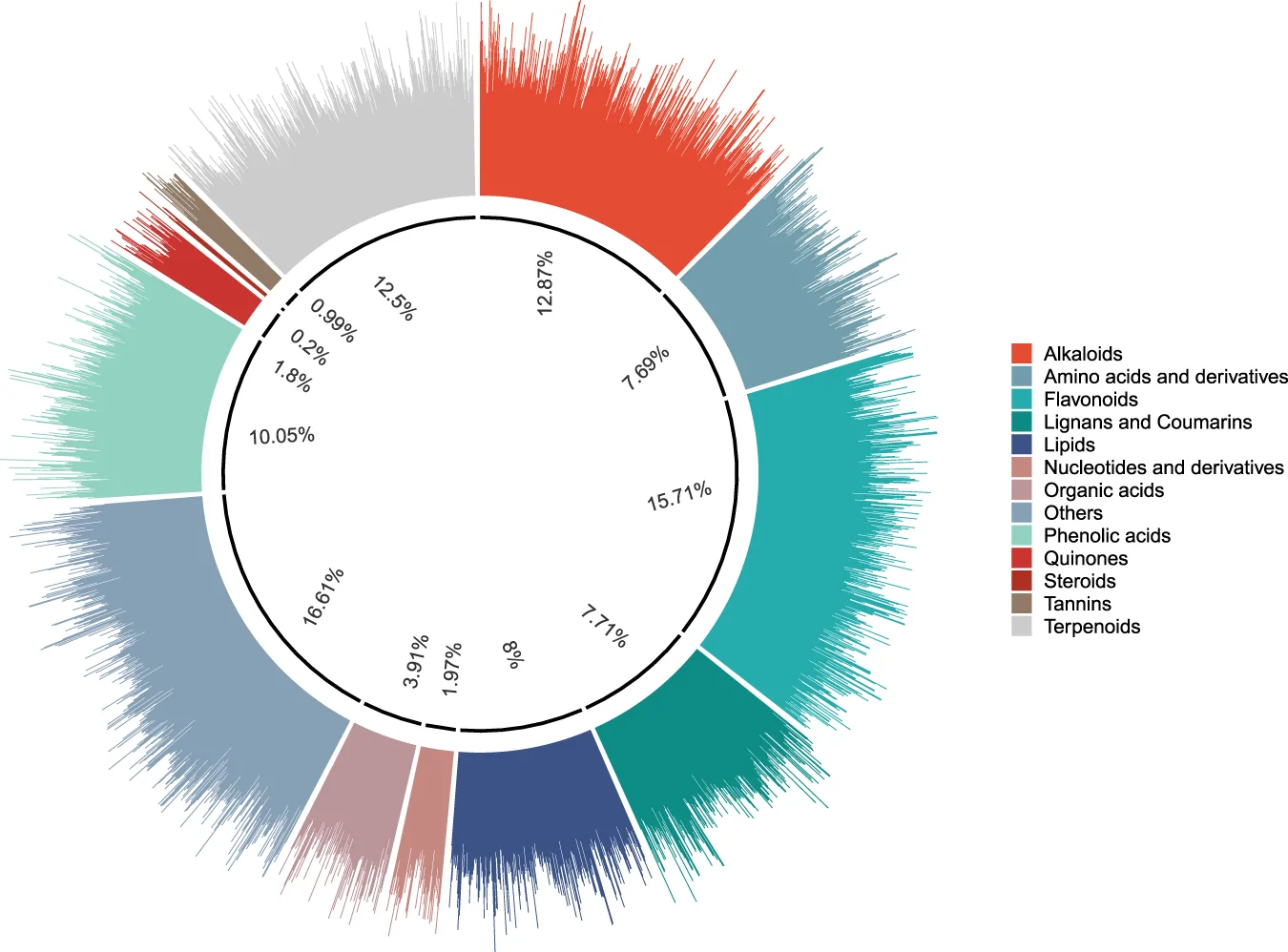
Chemical class distribution of compounds identified in the SJ extract.

A total of 3,552 compounds were identified in the SJ extract and classified into 12 major chemical classes (Figure 2). Flavonoids and alkaloids were the most abundant classes, accounting for 15.71% and 12.87% of the total identified compounds, respectively.

Among the identified compounds, 10 compounds were detected in 100% of the experimental samples with relatively high abundances (ranking within the top 100) and were selected as representative compounds for detailed presentation (Table 2). Among these, five were assigned MSI Level 1 identification confidence, including two phenolic acids (cryptochlorogenic acid and neochlorogenic acid) and three alkaloids (13-methylpalmatrubine, dehydrocorydalmine, and groenlandicine). The remaining five compounds were annotated at MSI Level 2 (see Table 2 footnote for details). In the alkaloid class, isoquinoline alkaloids such as 13-methylpalmatrubine, dehydrocorydalmine, and groenlandicine were the predominant subclasses, along with 5-(2-aminoethyl)-8-hydroxyquinolin-2(1H)-one. The complete metabolomic dataset is provided in the Supplementary Material.

**TABLE 2:** List of 10 representative compounds identified in the SJ extract.

Number	Compounds	Class I (superclass)	Class II (subclass)	Formula
1	Cryptochlorogenic acid (4-O-Caffeoylquinic acid)	Phenolic acids	Caffeoylquinic acids	C_16_H_18_O_9_
2	13-Methylpalmatrubine	Alkaloids	Isoquinoline alkaloids	C_21_H_22_NO_4_^+^
3	Neochlorogenic acid	Phenolic acids	Caffeoylquinic acids	C_16_H_18_O_9_
4	4-O-Feruloylquinic acid	Phenolic acids	Feruloylquinic acids	C_17_H_20_O_9_
5	3-O-Feruloylquinic acid	Phenolic acids	Feruloylquinic acids	C_17_H_20_O_9_
6	Dehydrocorydalmine	Alkaloids	Isoquinoline alkaloids	C_20_H_20_NO_4_^+^
7	13,13α-Didehydro-9,10-dimethoxy-2,3-(methylenedioxy)-berbine	Alkaloids	Isoquinoline alkaloids	C_20_H_19_NO_4_
8	1-O-Feruloylquinic acid	Phenolic acids	Feruloylquinic acids	C_17_H_20_O_9_
9	5-(2-aminoethyl)-8-hydroxyquinolin-2(1H)-one	Alkaloids	Quinolinone alkaloids	C_11_H_12_N_2_O_2_
10	Groenlandicine	Alkaloids	Isoquinoline alkaloids	C_19_H_16_NO_4_^+^

Compounds were selected based on their detection frequency (100% in all samples), relatively high abundance, and chemical diversity. Identification confidence was assigned according to the Metabolomics Standards Initiative (MSI): compounds marked with * were identified at MSI Level 1 (authentic standard-matched retention time and MS/MS spectrum), while unmarked compounds were annotated at MSI Level 2 (high-confidence MS/MS spectral matching against an in-house library). The selection was based on detection frequency and abundance rather than class representation.

### 3.2 The efficacy and safety results of the regenerating skin ointment on wound healing

#### 3.2.1 Effects of SJ on wound healing and serum angiogenic factors in patients

A total of 104 patients were enrolled in this study. Ten patients dropped out, and 94 completed the trial. In the SJ group, 4 patients withdrew. Two were lost to follow-up due to long travel distance, and another two quit voluntarily. In the control group, six patients dropped out. One switched to other medications, two left for other cities, and three withdrew on their own. The overall dropout rate was 9.6%. Most participants were elderly and had limited mobility, but patient compliance remained satisfactory given the long follow-up period.

Compared with the rbFGF group, the SJ group showed a more significant promoting effect on wound healing. According to the statistics, after 2 weeks of treatment, the wound area of patients in the SJ group was (5.30, {2.05, 9.85} cm^2^; n = 48), while that of patients in the rbFGF group was (6.60, {2.48, 12.78} cm^2^; n = 46). After 4 weeks of treatment, the wound area of patients in the SJ group was (2.55, {0.93, 4.85} cm^2^; n = 48), and that of patients in the rbFGF group was (5.35, {1.20, 9.38} cm^2^; n = 46). The difference between the two groups was significant (*P < 0.05*). Comparing the wound reduction rates of the two groups, after 2 weeks of treatment, the wound reduction rate of patients in the SJ group was (34.63, {24.62, 47.38} %; n = 48), while that of patients in the rbFGF group was (15.78, {8.10, 34.75} %; n = 46). The difference between the two groups was significant (*P < 0.01*). After 4 weeks of treatment, the wound reduction rate of patients in the SJ group was (64.42, {53.39, 80.05} %; n = 48), and that of patients in the rbFGF group was (35.42, {25.03, 53.17} %; n = 46), and the difference between the two groups was significant (*P < 0.01*).

**FIGURE 3:**
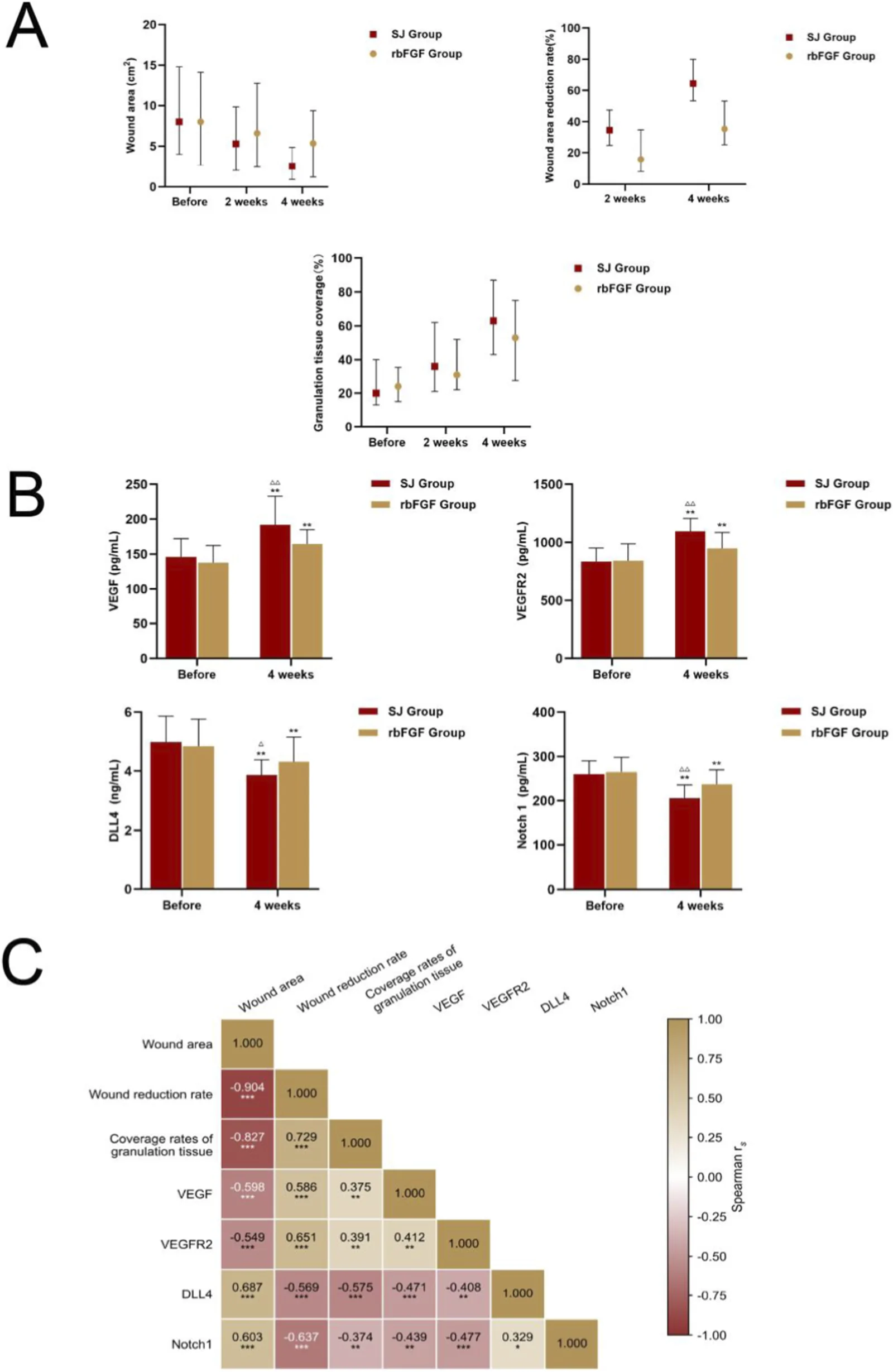
Effects of SJ on diabetic foot ulcer healing. **(A)** Wound area and epithelialization were measured using the Kusthe Ekare Insight insight 3D wound measurement recording system (V1.7.5). Measure the wound area, the rate of wound reduction, and the degree of epithelialization. The rate of wound reduction is calculated as the percentage change in area based on the baseline value. **(B)** Serum VEGF, VEGFR2, Notch1, and DLL4 levels were detected by ELISA. **P < 0.01 (SJ Group pre-treatment vs. post-treatment); ^*△*^ *P < 0.05* and ^*△△*^ *P < 0.01* (SJ Group vs. rbFGF Group). **(C)** Spearman correlation heatmap of wound healing indicators and serum-related factors. *P < 0.05, **P < 0.01, ***P < 0.001 (The values in the figure represent the Spearman rank correlation coefficient r_s_. The diagonal represents the correlation of the variables themselves).

Comparing the coverage rates of granulation tissue in the two groups, after 2 weeks of treatment, the coverage rate of granulation tissue on the wound surface of patients in the SJ group was (36.00, {21.00, 62.00} %, n = 48), while that of patients in the rbFGF group was (31.00, {22.00, 52.00} %, n = 46). After 4 weeks of treatment, the coverage rate of granulation tissue on the wound surface of patients in the SJ group was (63.00, {43.00, 87.00} %, n = 48), and that of patients in the rbFGF group was (53.00, {27.50, 75.00} %, n = 46). There was a significant difference between the two groups (*P < 0.01*) (Figure 3A).

The expression levels of angiogenic factors in the serum of the two groups of patients were compared. The results showed that after 4 weeks of treatment, when comparing the expression levels of VEGF between the two groups, the expression level of patients in the SJ group (191.71 ± 41.06 pg/mL) was higher than that of the rbFGF group (164.47 ± 20.45 pg/mL), and the difference was significant (*P < 0.01*). When comparing the expression levels of VEGFR2 between the two groups, the expression level of patients in the SJ group (1,095.94 ± 110.08 pg/mL) was higher than that of the rbFGF group (948.94 ± 136.95 pg/mL), and the difference was significant (*P < 0.01*). When comparing the expression levels of DLL4 between the two groups, the expression level of patients in the SJ group (3.86 ± 0.53 ng/mL) was lower than that of the rbFGF group (4.32 ± 0.84 ng/mL), and the difference was significant (*P < 0.01*). When comparing the expression levels of Notch1 between the two groups, the expression level of patients in the SJ group (205.75 ± 29.71 pg/mL) was lower than that of the rbFGF group (237.34 ± 32.32 pg/mL), and the difference was significant (*P < 0.01*) (Figure 3B).

Spearman’s rank correlation analysis was adopted in this study to analyze the correlations between wound healing indicators and serum biomarkers in patients after 4 weeks of treatment with Shengji Ointment. The values in the figure represent the Spearman rank correlation coefficient r_s_. Wound area was significantly negatively correlated with wound reduction rate and granulation tissue coverage rate *(P < 0.001)*.

Wound area exhibited a significant negative correlation with the expression levels of VEGF and VEGFR2 *(P < 0.001)*, whereas it was significantly positively correlated with DLL4 and Notch1 expression *(P < 0.001)*. The wound reduction rate was significantly positively associated with VEGF and VEGFR2 expression *(P < 0.001)*, while showing a significant negative correlation with DLL4 and Notch1 expression *(P < 0.001)*. Coverage rates of granulation tissue was significantly positively correlated with VEGF *(P = 0.009)* and VEGFR2 expression *(P = 0.006)*. By contrast, this indicator presented significant negative correlations with DLL4 *(P < 0.001)* and Notch1 expression *(P = 0.009)* (Figure 3C).

#### 3.2.2 Safety evaluation index values of two groups of patients

No significant differences were observed in CBC, liver function, renal function, and blood glucose between the SJ group and control group at baseline and 4 weeks after treatment (*P > 0.05*). CBC included white blood cells (WBC), red blood cells (RBC), platelets (PLT), and hemoglobin (Hb). Liver function included alanine transaminase (ALT), aspartate transaminase (AST), total protein (TP), and albumin (ALB). Renal function included serum creatinine (Cr) and blood urea nitrogen (BUN).No adverse events such as pruritus, burning sensation, or aggravated pain were observed during the observation period. The evaluation results of the two groups of safety indicators are presented in the Supplementary Material.

### 3.3 Effect of SJ on diabetic wound healing in rats

#### 3.3.1 Wound morphology, histology, and healing kinetics

**FIGURE 4:**
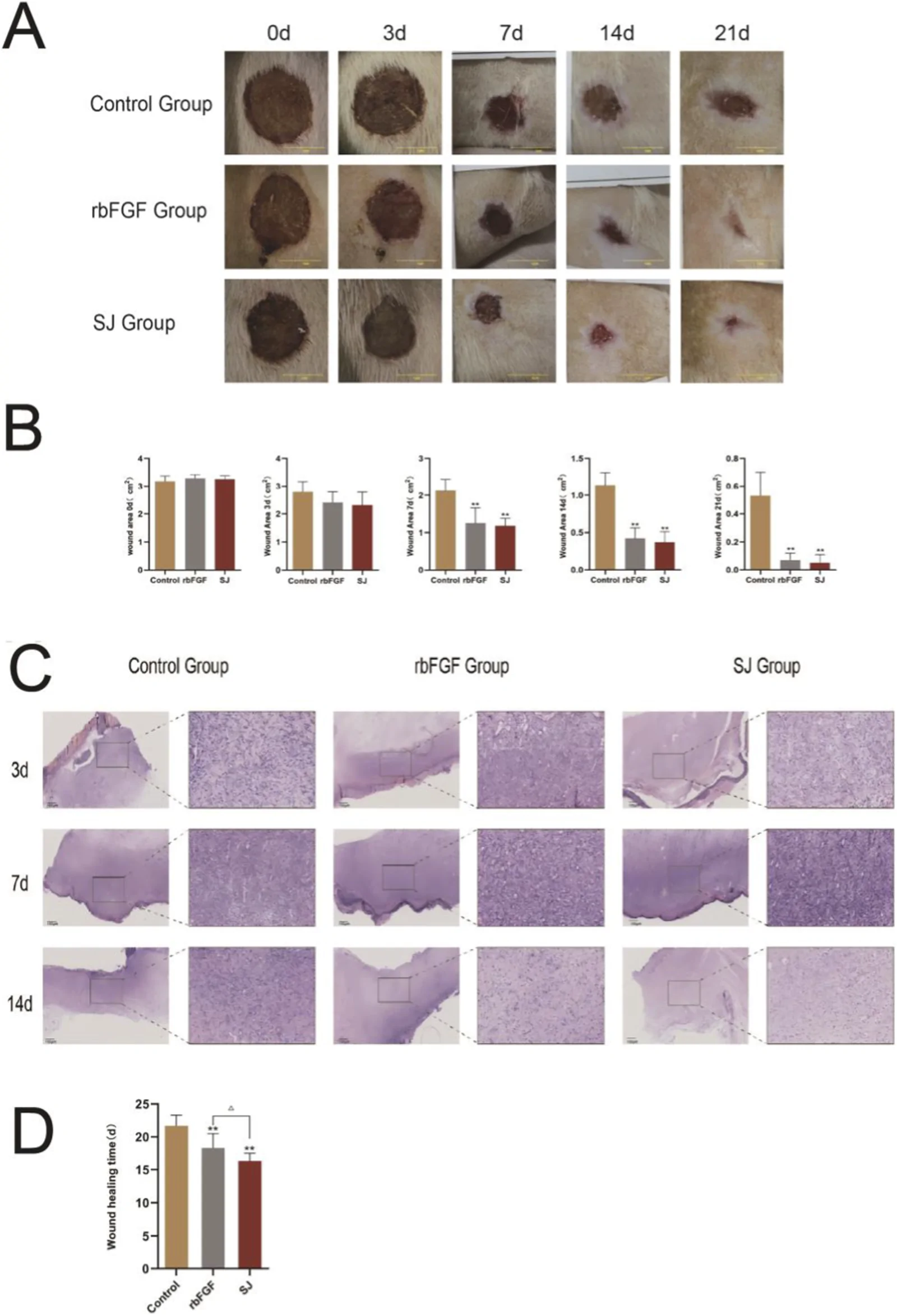
Effect of SJ on wound healing in diabetic rats. **(A)** Representative wound images from the SJ, rbFGF and Control groups at days 0, 3, 7, 14, and 21 post-treatments. **(B)** Wound area measurements at days 0, 3, 7, 14, and 21 post-treatments. **(C)** HE staining of wound tissues from all groups at days 3, 7, and 14 post-treatments. **(D)** Wound healing time from all groups. **P < 0.01 (SJ or rbFGF group vs. Control group); ^*△*^ *P < 0.05* (SJ group vs. rbFGF group).

On the seventh day post-treatment, the wound area in the SJ group measured (1.18 ± 0.33 cm^2^;n = 6), while that in the rbFGF group was (1.34 ± 0.30 cm^2^; n = 6), both demonstrating a significant reduction compared to the Control Group (2.12 ± 0.13 cm^2^; n = 6). By the 14th day, the wound areas further decreased to (0.37 ± 0.23 cm^2^; n = 6) in the SJ group and (0.58 ± 0.17 cm^2^; n = 6) in the rbFGF group, again showing significant reduction relative to the Control Group (1.14 ± 0.15 cm^2^; n = 6). At the 21st day, the wound areas were recorded as (0.05 ± 0.06 cm^2^; n = 6) for the SJ group and (0.12 ± 0.17 cm^2^; n = 6) for the rbFGF group, both markedly smaller than that of the Control Group (0.52 ± 0.17 cm^2^; n = 6) (Figures 4A,B).

On the third day post-treatment, acute inflammatory response intensified, predominantly characterized by neutrophil infiltration with minor lymphocyte involvement. By the seventh day, the infiltration of both neutrophils and lymphocytes subsided earlier in the SJ group compared to the control group. In contrast to the control, the SJ group exhibited advanced tissue remodeling, featuring mature collagen deposition, capillary formation, and squamous epithelial regeneration (Figure 4C).

Wounds in the SJ group achieved complete healing in 20.67 ± 0.94 days (n = 6), significantly faster than both the rbFGF group (22.33 ± 0.7 days; n = 6) and the control group (25.17 ± 1.07 days; n = 6) (Figure 4D).

#### 3.3.2 Expressions of inflammatory factors in rat wounds

At days 3, 7, 14, and 21 post-treatments, the pro-inflammatory factors IL-6 and TNF-αexpressions were significantly lower in the SJ group and rbFGF group compared to the control group. Furthermore, the SJ group showed a more pronounced reduction in IL-6 and TNF-α than the rbFGF group.

**FIGURE 5:**
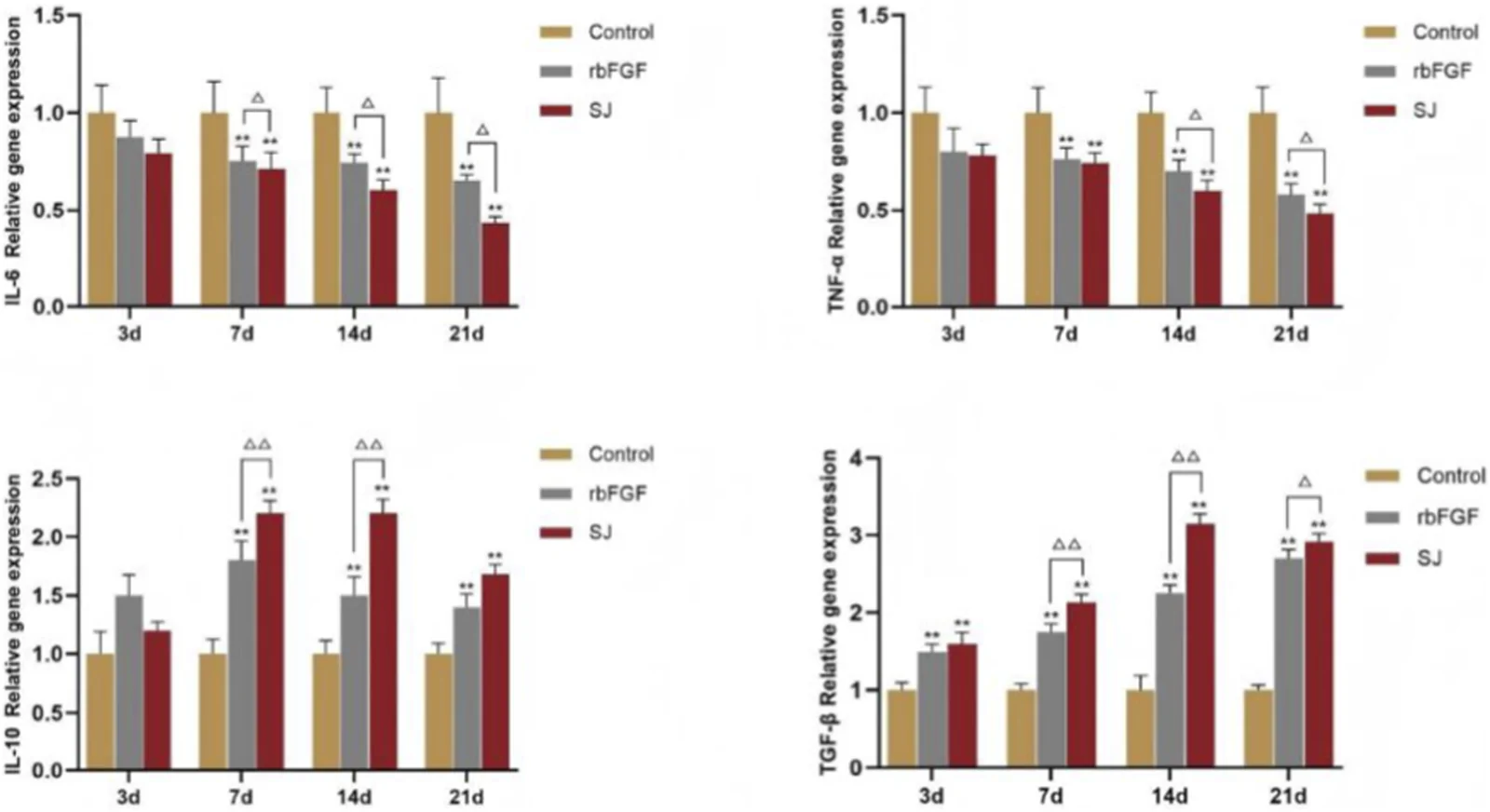
Gene expression of inflammatory factors in rat wounds. Pro-inflammatory factors IL-6 and TNF-α, and anti-inflammatory factors IL-10 and TGF-β were measured in rat wounds at days 3, 7, 14, and 21 post-treatments by real-time PCR. *P < 0.05, **P < 0.01 (SJ or rbFGF group vs. Control group). ^Δ^ *P < 0.05* and ^ΔΔ^ *P < 0.01* (SJ group vs. rbFGF group).

In contrast, the anti-inflammatory factors IL-10 and TGF-β gradually increased in the SJ group from day 3, reaching higher levels than both rbFGF group and Control groups (Figure 5).

#### 3.3.3 Effect of SJ on collagen fiber formation and expressions of MMP-2 and MMP-9 in rat wounds

**FIGURE 6:**
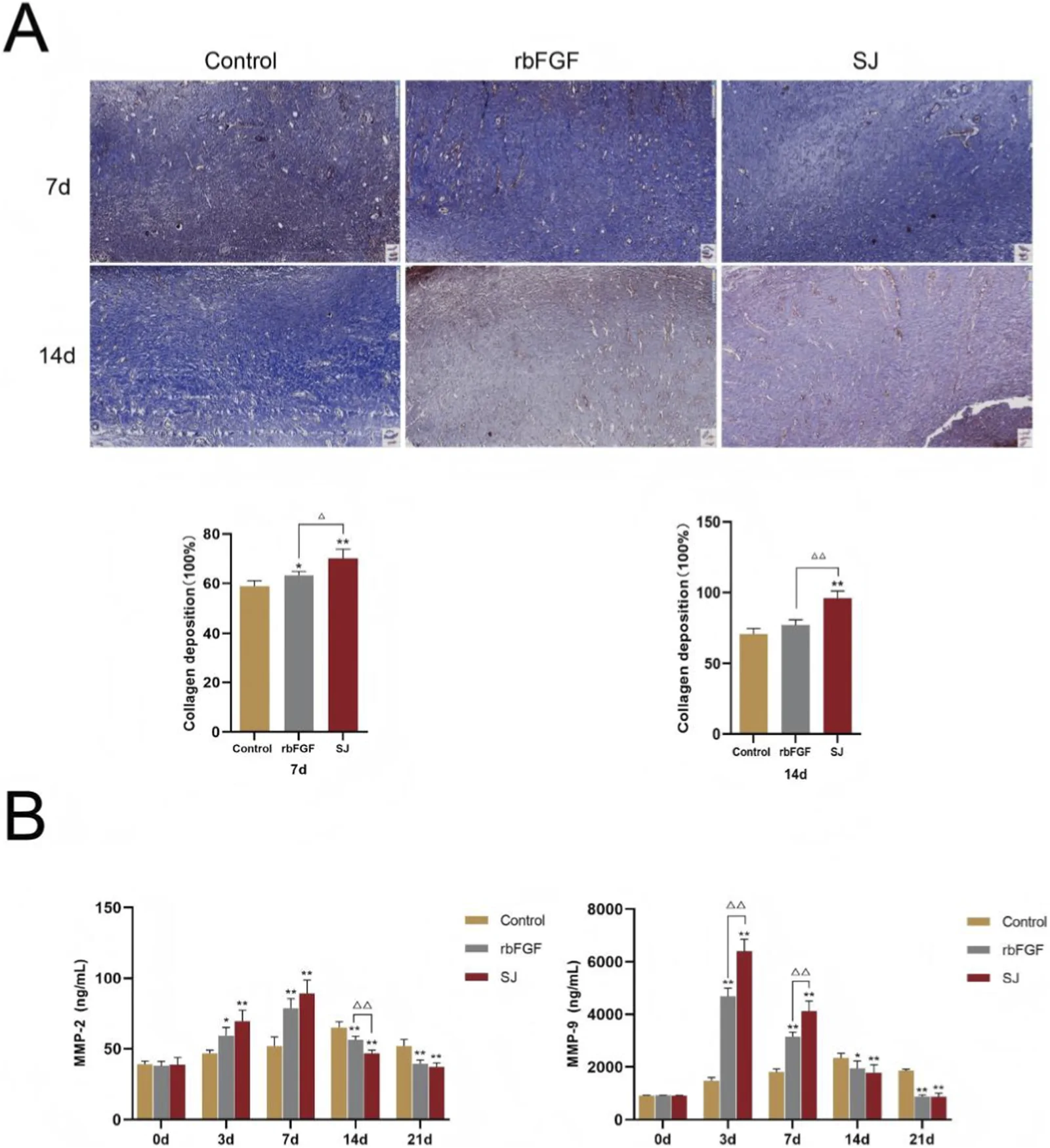
Collagen fiber morphology and MMP-2 and MMP-9 expression in rat wounds. **(A)** Masson Trichrome staining of rat wounds from three groups at days 7 and 14 post-treatment and Proportion of collagen deposition. **(B)** Protein expressions of MMP-2 and MMP-9 in rat wounds from three groups at days 3, 7, 14, and 21 detected by ELISA. *P < 0.05 and **P < 0.01 (SJ group vs. control group). ^*△*^ *P < 0.05* and ^*△△*^ *P < 0.01* (SJ group vs. rbFGF group).

Masson’s trichrome staining revealed that SJ treatment significantly increased fibroblast proliferation and collagen formation. At day 7 post-treatment, collagen fibres were thicker and irregularly arranged, whereas by day 14, collagen fibres exhibited dense and parallel alignment, compared with the Control and rbFGF groups (Figure 6A).

The protein expression of MMP-2 progressively increased from day 0 to day 7 post-treatment, peaking at day 7. The SJ group demonstrated higher MMP-2 levels than the Control group. Subsequently, the expression decreased from day 7 to day 14, significantly lower in the SJ group than in the Control group. MMP-9 expression in the SJ group peaked at seven times the baseline level (day 0). After an initial 3-day period, levels gradually decreased, falling below both the Control and rbFGF groups by day 14, and returning to baseline by day 21 (Figure 6B).

#### 3.3.4 Expression of VEGF and VEGFR2 in rat wounds

**FIGURE 7:**
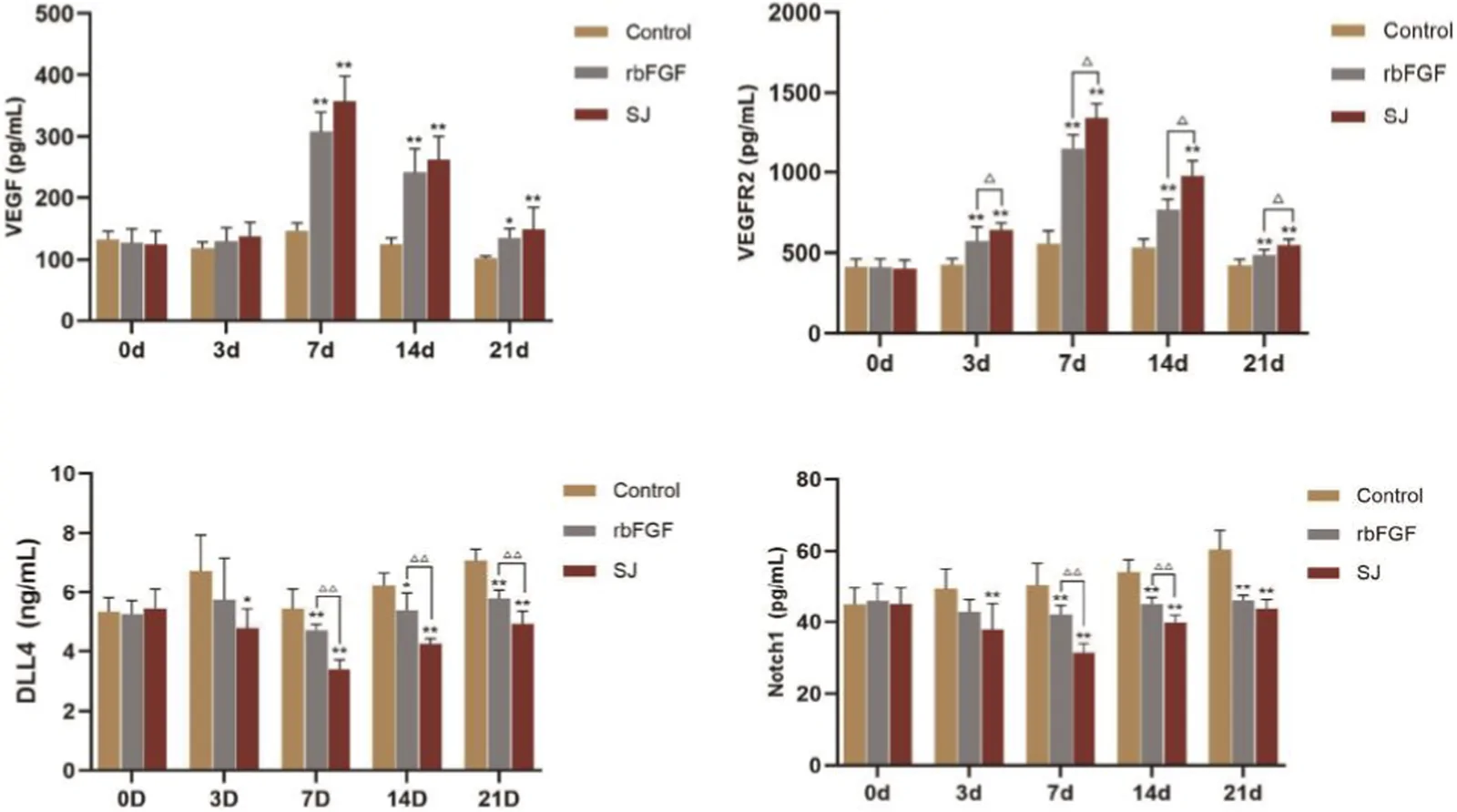
Expressions of VEGF, VEGFR2, DLL4, and Notch1 at days 3, 7, 14, and 21 in rat wounds from all groups. *P < 0.05, **P < 0.01 (SJ group or rbFGF group vs. Control group); ^△^ *P < 0.05*, ^△△^ *P < 0.01* (SJ group vs. rbFGF group).

In Figure 7, VEGF expression in rat wounds peaked at day 7 in all groups, followed by a gradual decrease as healing progressed. Throughout the entire observation period, the VEGF levels in the SJ group and the rbFGF group remained relatively high. On the seventh day, the expression level in the SJ group was (350.70 ± 40.89; n = 6), that in the rbFGF group was (307.83 ± 33.05; n = 6), and that in the Control group was (146.17 ± 12.73; n = 6). On the 14th day, the expression level in the SJ group was (262.67 ± 37.87; n = 6), that in the rbFGF group was (242.67 ± 37.87; n = 6), and that in the Control group was (124.19 ± 10.94; n = 6). On the 21st day, the expression level in the SJ group was (149.64 ± 35.11; n = 6), that in the rbFGF group was (135.72 ± 14.88; n = 6), and that in the Control group was (102.87 ± 3.36; n = 6). On the seventh, 14th, and 21st days, compared with the Control group, the SJ group had the highest expression level, followed by the rbFGF group, and the differences were statistically significant *(P < 0.05, P < 0.01).*

VEGFR-2 expression was consistent with VEGF. Both the SJ and rbFGF groups maintained elevated VEGFR-2 levels at all time points, demonstrating statistically significant differences compared to the Control group on day 3, 7, 14, and 21. Relative to the rbFGF group, the SJ group exhibited higher expression levels with statistically significant differences *(P < 0.05, P < 0.01)*. On day 3, VEGFR2 levels were (641.10 ± 42.77; n = 6) in the SJ group, (572.34 ± 86.04; n = 6) in the rbFGF group, and (424.77 ± 39.46; n = 6) in the Control group. On day 7, levels reached (1,336.17 ± 96.81; n = 6) in the SJ group, (1,147.92 ± 83.35; n = 6) in the rbFGF group, and (556.84 ± 78.53; n = 6) in the Control group. On day 14, expression levels were (977.11 ± 93.68; n = 6) in the SJ group, (768.83 ± 63.23; n = 6) in the rbFGF group, and (532.56 ± 52.53; n = 6) in the Control group. On day 21, levels were (546.96 ± 37.02; n = 6) in the SJ group, (483.99 ± 34.35; n = 6) in the rbFGF group, and (421.45 ± 38.50; n = 6) in the Control group.

Regarding the expression levels of DLL4, both the SJ group and the rbFGF group exhibited an initial decline followed by an upward trend. On day 3, the expression level in the SJ group was (4.76 ± 0.67; n = 6), that in the rbFGF group was (5.75 ± 1.41; n = 6), and that in the Control group was (6.73 ± 1.20; n = 6). A statistically significant difference was observed between the SJ group and the Control group (P < 0.05). On day 7, the expression levels were (3.38 ± 0.33; n = 6) in the SJ group, (4.70 ± 0.20; n = 6) in the rbFGF group, and (5.43 ± 0.67; n = 6) in the Control group. On day 14, the levels were (4.25 ± 0.18; n = 6), (5.39 ± 0.58; n = 6), and (6.23 ± 0.44; n = 6), respectively. On day 21, the expression levels reached (4.91 ± 0.44; n = 6) in the SJ group, (5.78 ± 0.29; n = 6) in the rbFGF group, and (7.09 ± 0.37; n = 6) in the Control group. At days 7, 14, and 21, both the SJ and rbFGF groups showed significantly lower expression levels compared to the Control group, with the differences being statistically significant *(P < 0.05, P < 0.01)*.

Finally, the expression level of N-cadherin (N-cad) exhibited a progressive upward trend in both the SJ and rbFGF groups throughout the entire observation period, whereas the SJ group demonstrated an initial decline followed by a subsequent increase. On day 3, the expression levels were (37.81 ± 7.34; n = 6) in the SJ group, (42.84 ± 3.46; n = 6) in the rbFGF group, and (49.24 ± 5.89; n = 6) in the Control group, with a statistically significant difference between the SJ and Control groups *(P < 0.01)*. On day 7, the expression levels were (31.52 ± 2.40; n = 6) in the SJ group, (42.12 ± 2.48; n = 6) in the rbFGF group, and (50.64 ± 5.98; n = 6) in the Control group. On day 14, the expression levels were (39.78 ± 2.09; n = 6) in the SJ group, (45.06 ± 1.83; n = 6) in the rbFGF group, and (54.23 ± 3.43; n = 6) in the Control group, with statistically significant differences observed between the SJ group and both the rbFGF and Control groups on days 7 and 14 *(P < 0.01)*. On day 21, the expression levels were (43.73 ± 2.57; n = 6) in the SJ group, (46.10 ± 1.40; n = 6) in the rbFGF group, and (60.43 ± 5.30; n = 6) in the Control group, where the SJ group exhibited significantly lower expression levels compared to the other two groups *(P < 0.01)* (Figure 7).

## 4 Discussion

Diabetic wound healing, characterized by persistent inflammation, impaired angiogenesis, and dysfunctional cellular responses, remains a major clinical challenge due to its complex pathophysiology. This challenge is most evident in DFUs, which carry high risks of infection, recurrence, and amputation, contributing substantially to patient morbidity, mortality, and healthcare costs (Olamoyegun et al., 2026; Pavithra and Kaliyaperumal, 2026).

Normal wound healing includes hemostasis, inflammation, proliferation and remodeling. Inflammation is a core physiological response throughout wound healing, and the dynamic change of inflammatory factors directly regulates the progression of wound repair, including tissue repair, angiogenesis and collagen deposition (Liu et al., 2026; Lopes et al., 2024). During the early inflammatory stage of wound repair, pro-inflammatory cytokines such as TNF-α and IL-6 are highly expressed to eliminate wound pathogens and necrotic debris. With the gradual resolution of inflammation, the levels of anti-inflammatory factors and growth factors including IL-10 and TGF-β increase correspondingly, which facilitates cell proliferation, angiogenesis and tissue remodeling, and ultimately promotes wound healing (Beaziz et al., 2026). Persistent abnormal inflammatory responses are the key cause of delayed healing in diabetic wounds (Wolf et al., 2021). Long-term hyperglycemia can disrupt the inflammatory homeostasis of wound microenvironment, sustain the overexpression of pro-inflammatory factors, and inhibit the secretion of repair-related growth factors. This abnormal inflammatory state further suppresses the proliferation and migration of fibroblasts and endothelial cells, thereby hindering the repair process of diabetic wounds (Baltzis et al., 2014). The important members of the matrix metalloproteinase (MMP) family, MMP-2 and MMP-9, are involved in collagen degradation and extracellular matrix (ECM) remodeling. High MMP-9 expression is thought to be related to the infiltration of inflammatory cells and the clearance of necrotic tissue in the early stage of wound healing, while MMP-2 promotes fibroblast migration, angiogenesis, and collagen remodeling in the middle and late stages of wound healing. The balance between these two is crucial for the recovery process, and corresponding intervention measures need to be adjusted according to the recovery stage and type of injury (Raziyeva et al., 2021; Lazaro et al., 2016).

Angiogenesis is essential for wound repair. The VEGF/VEGFR pathway promotes vascular sprouting. In a high-glucose environment, overactivated DLL4/Notch1 suppresses VEGF/VEGFR signaling, disrupting angiogenesis and hindering diabetic foot ulcer healing (White et al., 2021; Chi et al., 2026; Johnson and Wilgus, 2014; Wang et al., 2018; Gao et al., 2022). In diabetic foot ulcers, sustained hyperglycemia disrupts this process: it induces chronic inflammation, damages vascular endothelium and suppresses angiogenesis. The disturbed microenvironment hinders granulation tissue growth and collagen remodeling, leading to intractable wounds (Dawi et al., 2025).

Shengji Ointment, a preparation composed of natural ingredients, has long been used in ancient China for the treatment of chronic refractory wounds. Clinical practice has verified that it can effectively accelerate wound healing. It facilitates the transition of chronic wounds from the inflammatory stage to the proliferative stage and promotes the growth of granulation tissue, which has gained wide recognition in clinical application.

Among the identified phenolic acids, cryptochlorogenic acid (4-O-caffeoylquinic acid) has been reported to attenuate LPS-induced inflammatory responses and oxidative stress in RAW 264.7 macrophages by upregulating the Nrf2/HO-1 signaling pathway, as evidenced by dose-dependent inhibition of NO, TNF-α, and IL-6 production (Zhao et al., 2020). Neochlorogenic acid has been shown to suppress NF-κB-mediated NLRP3 inflammasome activation, thereby reducing endothelial injury in diabetic models (Tsai et al., 2026), and to alleviate allergic airway inflammation through HO-1 upregulation (Cheng et al., 2025). Additionally, 4-O-feruloylquinic acid has been identified as an active component that modulates PAR-2 signaling and promotes Ca^2+^ influx in keratinocytes (Choi et al., 2020), while 3-O-feruloylquinic acid exhibited antioxidant and anti-inflammatory activities by inhibiting NO production in LPS-stimulated RAW 264.7 cells (Ao et al., 2022). These findings suggest that the phenolic acid constituents in SJ, particularly the caffeoylquinic and feruloylquinic acid derivatives, may collectively contribute to the anti-inflammatory and antioxidant properties of the formulation.

Among the isoquinoline alkaloids identified, 13-methylpalmatrubine has been reported to inhibit proliferation, invasion, and epithelial-mesenchymal transition in non-small cell lung cancer cells, and to induce apoptosis through blockade of EGFR signaling and activation of MAPK pathways (Chen et al., 2016; Wu et al., 2022). Dehydrocorydalmine, another isoquinoline alkaloid present in SJ, has demonstrated gastroprotective and antibacterial activities against *Helicobacter pylori* (Wu et al., 2023). Groenlandicine, a protoberberine-type isoquinoline alkaloid structurally related to berberine, falls within a compound class that has been extensively investigated for wound healing applications, with berberine itself showing effects on diabetic wound repair through multiple mechanisms (Jin et al., 2026; Fang et al., 2026). Although the specific contributions of these isoquinoline alkaloids to wound healing have not been directly established, their documented anti-inflammatory, antimicrobial, or cell-proliferation-modulating activities may be relevant to the multifaceted pathological processes of diabetic wound repair. However, it should be acknowledged that, based on the current literature, no direct evidence has confirmed the involvement of quinolinone alkaloids in wound repair, inflammation, or angiogenesis. Nevertheless, the high-confidence identification of these alkaloids in the SJ extract warrants further investigation. These compounds may contribute to the observed therapeutic effects, either individually or through multi-target combined effects with other constituents; these possibilities remain to be experimentally verified.

In the present study, we measured the serum levels of angiogenesis-related factors in patients ‘peripheral blood. Consistent with prior wound repair research (Solly et al., 2026), higher expression of VEGF and VEGFR2 was associated with smaller wound areas and greater wound reduction rates (Li L. et al., 2025). As a canonical pro-angiogenic mediator, VEGF binds and activates VEGFR2 to drive endothelial cell proliferation, migration and neovascularization. This process delivers oxygen and nutritional substrates to the wound microenvironment, facilitating tissue regeneration and wound contraction. Conversely, elevated levels of DLL4 and Notch1 are associated with larger wound areas, indicating that the overexpression of these two factors inhibits wound healing. Notably, Shengji Ointment can significantly downregulate the expression of these two mediators. Upon binding to Notch1, DLL4 restrains excessive activation of vascular endothelial cells and suppresses aberrant proliferation and remodeling of nascent blood vessels to maintain angiogenic homeostasis (Lv et al., 2025; Zhang et al., 2025). Collectively, the progression of wound healing relies heavily on the dynamic balance between pro-angiogenic signals mediated by VEGF/VEGFR2 and anti-angiogenic signals driven by DLL4/Notch1.

Clinical and animal data were consistent, SJ significantly enhanced rat diabetic wound healing through stage-specific modulation of inflammatory and angiogenic pathways. In the inflammatory stage, SJ suppressed the expression of pro-inflammatory factors (e.g., IL-6,TNF-α), but promoted the production of anti-inflammatory factors (e.g., IL-10,TGF-β) in the proliferation stage. SJ significantly promoted fibroblast proliferation and collagen formation. It increased VEGF and VEGFR2 levels, and decreased DLL4 and Notch1 expression, these molecular changes emerged on Days 3 and 7, before obvious wound closure,In line with published reports (Zheng et al., 2019). SJ upregulated the expression of VEGF and VEGFR2, and downregulated the expression of DLL4 and Notch1 in the inflammatory stage, facilitating the budding and migration of stem cells and promoting capillary formation. Later, the DLL4 and Notch1 expression increased and VEGF and VEGFR2 expression decreased, promoting stem cells differentiation, capillary maturation, and lumen formation.

Compared with rbFGF, the therapeutic effect of SJ is significantly better. The reasons for this might lie in the following points. rbFGF is a single component and can only promote cell proliferation, while SJ is a multi-component and multi-target herbal preparation that can simultaneously inhibit excessive inflammation, restore the balance of vascularization, promote collagen deposition and orderly maturation, and accelerate extracellular matrix remodeling. These combined effects create a favorable microenvironment for continuous and stable wound repair, and a single growth factor often cannot correct the persistent inflammatory imbalance and insufficient vascular maturation problems in diabetic wounds. Compared with single growth factor therapy, when benchmarked against single growth factor therapy, SJ exhibits an integrated regulatory pattern derived from the combined actions of multiple active constituents enabling more comprehensive modulation of physiological processes. This multi-target regulatory mode can simultaneously intervene in multiple pathological links of diabetic wounds, including inflammation, vascularization, and matrix remodeling, thereby achieving a more durable and thorough repair effect.

This study has several limitations. First, only clinical correlation analyses of wound healing status and angiogenesis-related factors were conducted in the clinical trial. Such analyses can merely identify associations between variables, yet cannot establish causal relationships or upstream and downstream regulatory mechanisms linking these factors to wound healing indices. Second, serial dynamic measurements at multiple time points were absent, which prevented comprehensive characterization of the temporal expression patterns of these factors throughout wound repair.

As for the rat experiments, the STZ-induced diabetic wound model cannot fully recapitulate the complicated pathological features of clinical diabetic foot ulcers, such as peripheral neuropathy and persistent infection. Although multiple detection approaches including RT-qPCR, ELISA, H&E staining and Masson’s trichrome staining were adopted for comprehensive analysis, all experiments were performed *in vivo* without further validation at the cellular level. In addition, this work only explored the overall therapeutic efficacy of Shengji Ointment and general correlations between its major components and targeted signaling pathways. The specific regulatory mechanisms of individual active ingredients remain to be elucidated in future investigations.

## 5 Conclusion

This study demonstrates that Shengji Ointment significantly accelerates wound healing in both clinical subjects with diabetic foot ulcers and a diabetic rat wound model. SJ suppresses excessive inflammatory responses by downregulating pro-inflammatory factors while upregulating anti-inflammatory mediators. Furthermore, SJ promotes angiogenesis as well as the ordered arrangement and maturation of collagen fibers, and such therapeutic outcomes may be achieved by regulating the balance of the DLL4/Notch1-VEGF/VEGFR2 signaling pathway. Together, these effects contribute to enhanced tissue repair and rapid wound closure. Collectively, our findings indicate that SJ represents a promising plant-derived drug candidate for the clinical management of diabetic wounds. Future studies will be directed toward validating these observations through mechanistic investigations of Notch signaling pathway modulation, as well as conducting in-depth pharmacological characterization of the bioactive constituents identified in the SJ extract to elucidate their individual contributions to the observed therapeutic effects.

## Data Availability

The original contributions presented in the study are included in the article/Supplementary Material, further inquiries can be directed to the corresponding authors.
