# Supplementary material for: Shengji ointment accelerates diabetic foot ulcer wound healing by regulating inflammatory microenvironment
Source: Front Pharmacol. 2026 Sep 3;17:1855791. doi: 10.3389/fphar.2026.1855791 (PMC13582591; doi:10.3389/fphar.2026.1855791)
Supplement: Supplementary file 1 [file Table2.docx]

Tab. 2 Safety evaluation index values of two groups of patients

| Indicators of evaluation | Items of inspection | Group | Before treatment | After 4 weeks of treatment | P |
| --- | --- | --- | --- | --- | --- |
| Blood routine test | White cells | SJ | 6.94（5.67，8.42） | 6.86（6.15，7.98） | 0.950 |
|  |  | Control | 7.28（6.23，8.66） | 7.10（5.47，8.23） | 0.217 |
|  | Red blood cells | SJ | 4.14±0.57 | 4.33±0.51 | 0.087 |
|  |  | Control | 4.10±0.56 | 4.16±0.57 | 0.660 |
|  | Blood platelets | SJ | 277.21±61.82 | 255.73±55.47 | 0.115 |
|  |  | Control | 271.04±92.14 | 251.91±90.28 | 0.322 |
|  | Hemoglobin | SJ | 123.31±19.23 | 129.67±18.03 | 0.102 |
|  |  | Control | 121.46±17.87 | 123.57±18.14 | 0.580 |
| Renal function | Creatinine | SJ | 64.05（54.10，77.65） | 68.40（56.15，78.95） | 0.112 |
|  |  | Control | 75.12（60.73，102.40） | 67.35（58.70，90.25） | 0.477 |
|  | Urea nitrogen | SJ | 5.21（4.26，6.23） | 5.03(3.77，5.48) | 0.093 |
|  |  | Control | 6.20（4.63，8.61） | 6.04（4.45，8.19） | 0.743 |
|  | Alanine aminotransferase | SJ | 12.50(10.25，21.75) | 12.50(9.25，20.00) | 0.808 |
|  |  | Control | 14.00(10.00，29.00) | 12.50(8.00，20.00) | 0.269 |
|  | Aspartate aminotransferase | SJ | 15.50(12.00，19.00) | 14.50(12.00，20.00) | 0.886 |
|  |  | Control | 16.00(11.75，18.00) | 12.00(10.00，17.25) | 0.063 |
| Liver function | Total protein | SJ | 68.36±7.04 | 71.16±7.01 | 0.057 |
|  |  | Control | 69.09±7.24 | 70.13±6.55 | 0.467 |
|  | Albumin | SJ | 39.08±6.09 | 41.45±6.38 | 0.069 |
|  |  | Control | 38.95±5.61 | 39.85±5.50 | 0.447 |
|  | Total bilirubin  Direct | SJ | 7.50（5.65，9.18） | 8.10（5.83，10.05） | 0.273 |
|  |  | Control | 7.65（5.38，9.23） | 7.70（5.95，10.35） | 0.395 |
|  | Bilirubin | SJ | 3.37±1.06 | 3.88±1.83 | 0.101 |
|  |  | Control | 3.05（2.48，4.70 | 3.20（2.58，4.25） | 0.737 |
| Blood glucose | －－ | SJ | 6.44±1.73 | 6.91±1.93 | 0.220 |
|  |  | Control | 6.71±1.84 | 6.87±1.66 | 0.677 |
